# Zoometric measures and their utilization in prediction of live weight of local goats in southern México

**DOI:** 10.1186/s40064-015-1424-6

**Published:** 2015-11-12

**Authors:** E. J. Dorantes-Coronado, Glafiro Torres-Hernández, O. Hernández-Mendo, R. Rojo-Rubio

**Affiliations:** Programa de Ganadería, Colegio de Postgraduados-Campus Montecillo, 56230 Montecillo, Edo. de México Mexico; Universidad Autónoma del Estado de México-Unidad Temascaltepec, Temascaltepec, Edo. De México Mexico

**Keywords:** Goats, Live weight, Zoometric measures, Prediction equations

## Abstract

Objectives of this study were: (a) to compare live weight (LW) and zoometric measures (ZM) of local goats in two locations, (b) to fit the best regression equation for goat LW prediction using ZM. LW, body length (BL), trunk length (TL), withers height (WH), hearth girth (HG), rump width (RW), rump length (RL), head length (HL), head width (HW), and ear length (EL) were measured in 318 Local does in Amatepec and Tejupilco, State of Mexico. Statistical methods included student’s “t” tests for comparison of means, and correlation, principal components (PC), and multiple linear regression analyses. To evaluate the goodness of fit for LW prediction models the R^2^ value was used as a criterion. Differences (*P* ≤ 0.05) were found between does of Amatepec and Tejupilco in LW, BL, TL, HG, RL, HL, HW, and EL. In Amatepec, LW was correlated with HG, BL, and HW (*P* ≤ 0.01), whereas in Tejupilco LW was correlated with HG, BL, TL, and HW (*P* ≤ 0.01). From the Amatepec measures 5 PC were extracted, and which in a multiple regression analysis explained 83.3 % of the total variance, whereas from Tejupilco 4 PC were extracted, and which in a multiple regression analysis explained 82.4 % of the total variance. The best regression model to predict doe LW in Amatepec included TL, HG, RW, and HW, whereas for Tejupilco the best model included BL, HG, HW, and EL. It is concluded that: (1) Amatepec does surpass those of Tejupilco in LW and most ZM, (2) there are reliable ZM for predicting LW of local does in both locations, HG, and HW being common measures for both populations.

## Background

It has been mentioned that breed characterization is a basic step to approach the sustainable use of animal genetic resource (Lanari et al. 2003). In the State of México, México, the region with the largest goat production is located in the rural municipalities of Amatepec and Tejupilco, with about 59 % of the total state goat production (Rebollar-Rebollar et al. 2012), with a predominance of local goats (Montaldo et al. 2010). The main product of goat farming in this region is meat; consequently, the trait of major interest for genetic improvement is live weight (LW). However, for goat producers in these locations it is very difficult to measure LW because most producers lack scales in order to weigh their animals, and sale prices depend entirely on live weight. Slippers et al. (2000) consider that visual estimates are too subjective; however, Otoikhian et al. (2008) indicate that it is feasible to achieve acceptable accuracy after having repeatedly estimated LW in goats by visual assessment. Nsoso et al. (2004) mentioned that it is possible to estimate LW in rural areas from the combination of zoometric measures and regression models, as a quick practice, of minimum cost and high reliability.

In Mexico, there is very little information regarding genetic and phenotypic variability of local goat populations, and even more about the efficiency of zoometric measures to estimate these parameters (Hernández Zepeda et al. 2002; Vargas et al. 2007). Objectives of the study were: (a) to compare LW and zoometric measures of local goats in two locations, and (b) to fit the best regression equation for LW prediction using zoometric measures.

## Methods

### Animals and study area

The present study was conducted in 17 goat production units (PU) of Amatepec and Tejupilco, located southwest of the State of Mexico, whose main characteristics are shown in Table 1. This study was conducted from February 2009 to October 2010, using 318 3-year-old local does. The management system is semi-extensive, with day grazing and night confinement. There are no regular health control practices and objective of the PU are breeding, sale of kids, and cull animals at weaning.

**Table 1:** Number of goats, climatic characteristics, altitude above sea level, and predominant forage species in Amatepec and Tejupilco, state of Mexico

Characteristics	Location	
	Amatepec	Tejupilco
Number of goats	142	176
Climate	Temperate sub-humid	Warm sub-humid
Precipitation (mm)^a^	1840	1200
Temperature (°C)^a^	22	27
Average altitude (masl)	1700	1200
Predominant forage species	*Paspalum notatum*	*Cynodon plectostachyus*
	*Sida rhombifolia*	*Brachiaria decumbens*
	*Ricinus comunnis*	*Andropogon gayanus*
	*Crescentia cujete*	*Gliricidia sepium*
	*Amaranthus hybridus*	*Leucaena esculenta*

^a^Annual averages. Source: INEGI (2008)

### Zoometric measures analyzed

LW (kg) of does was measured along with the following linear zoometric measures (cm): body length (BL), trunk length (TL), withers height (WH), hearth girth (HG), rump width (RW), rump length (RL), head length (HL), head width (HW), and ear length (EL). LW was measured using a hanging scale of 100 kg of capacity and 100 g of accuracy. Zoometric measures were performed with a measuring tape, a drill compass and a pediometer or a graduated measuring stick (Revidatti et al. 2007). Because pregnancy produces bias in some zoometric measures, especially in the regions of the chest and rump (Yakubu et al. 2011), only non-pregnant does were utilized in this study.

### Statistical methods

To test whether differences existed in means of LW and zoometric measures between does of Amatepec and Tejupilco student’s “t” tests for comparison of means (Steel and Torrie 1980) were performed. Means and standard deviations were obtained and the Pearson phenotypic correlation matrix was estimated for LW and zoometric measures.

In order to reduce the number of zoometric predictor measures (ZM) to estimate LW an analysis of principal components (APC) was carried out. This analysis allows transforming the original ZM in a new set of orthogonal variables (uncorrelated) called principal components (PC), which are a linear combination of the original variables (Johnson and Wichern 2007). In addition, APC of zoometric measures has been used as a tool in the assessment of animal body size and shape in goats (Okpeku et al. 2011). Upon APC, 9 PC were determined, and their variances, relative percentages and cumulative variances explained by the PC were obtained. The calculations were performed with the procedure PRINCOMP of SAS (SAS 2001).

For each location two multiple linear regression models were utilized to predict doe LW. The first, using as predictor variables the original ZM selected with the procedure STEPWISE of SAS (SAS 2001) based on the significance of their parameters (*P* **≤** 0.05), and the second, using as predictor variables the transformed values (“scores”) of the PC, that were selected based on the percentage of the variance that the PC explained. In both models the variables were standardized with mean 0 and variance 1, for facilitating their comparison.

The models have the following structure:

$$ {\text{LW }} = {\rm{\upbeta }}_{ 0} + {\rm{\upbeta }}_{\text{i}} {\text{X}}_{\text{i}} + \cdots + {\rm{\upbeta }}_{\text{n}} {\text{X}}_{\text{n}} + {{\epsilon }}\;\;\;\;\;\;{\text{(Model }}\; 1) $$

where LW is live weight, β_0_ is the intercept of the regression equation, β_i_ is the ith partial regression coefficient of the ith zoometric measure retained in the model (X_i_), β_n_ is the nth partial regression coefficient of the nth zoometric measure retained in the model (X_n_), ε is the random error.

$$ {\text{LW}} = {\rm{ \upgamma }}_{0} + {\rm{ \upgamma }}_{\text{i}} {\text{PC}}_{\text{i}} + \cdots + {\rm{\upgamma }}_{\text{n}} {\text{PC}}_{\text{n}} + {\rm{\epsilon }}\;\;\;\;{\text{(Model 2)}} $$

where LW is live weight, γ_0_ is the intercept of the regression equation, γ_i_ is the ith partial regression coefficient of scores of the ith principal component (PC_i_), γ_n_ is the nth partial regression coefficient of scores of the nth principal component (PC_n_), ε is the random error.

The criterion to evaluate goodness of fit of models used was based on the R^2^ value (Kleinbaum and Kupper 1978).

## Results

### Descriptive statistics

Table 2 shows descriptive statistics for LW and zoometric measures of goats from the two locations studied. Amatepec does were heavier, had a longer body, trunk, rump, head, and ear, and also a higher hearth girth than Tejupilco does.

**Table 2:** Least-squares means of live weight and zoometric measures of local goats in Amatepec and Tejupilco, state of Mexico

Variable	Amatepec (n = 142)		Tejupilco (n = 176)	
	Mean	SD	Mean	SD
LW (kg)	34.5 a	7.2	32.0 b	8.9
BL (cm)	104.5 a	8.4	98.9 b	9.6
TL (cm)	67.8 a	5.3	66.5 b	6.6
WH (cm)	66.2 a	4.9	66.4 a	5.4
HG (cm)	77.3 a	6.6	74.7 b	6.7
RW (cm)	13.7 a	2.0	14.0 a	1.8
RL (cm)	19.7 a	2.3	18.4 b	3.1
HL (cm)	20.8 a	2.1	20.2 b	2.4
HW (cm)	11.6 b	1.0	12.7 a	1.3
EL (cm)	18.3 a	2.6	17.3 b	2.9

a, b: different letters between rows differ (*P* ≤ 0.05)

*LW* live weight, *BL* body length, *TL* trunk length, *WH* withers height, *HG* hearth girth, *RW* rump width, *RL* rump length, *HL* head length, *HW* head width, *EL* ear length, *SD* standard deviation

### Phenotypic correlations

The matrix of phenotypic correlations between live weight and zoometric measures is shown in Table 3. The highest phenotypic correlations (*P* ≤ 0.01) were found between LW and HG (0.83), HG and TL (0.75), BL and TL (0.74), HG and WH (0.73), LW and BL (0.72), and LW and HG (0.71). Except for EL, LW was highly correlated (*P* ≤ 0.01) with all zoometric measures.

**Table 3:** Matrix of phenotypic correlations between live weight and zoometric measures of local goats in Amatepec (above main diagonal) and Tejupilco (below main diagonal), state of Mexico

	LW	BL	TL	WH	HG	RW	RL	HL	HW	EL
LW		0.63^a^	0.48^a^	0.49^a^	0.83^a^	0.52^a^	0.33^a^	0.40^a^	0.53^a^	0.00^ns^
BL	0.72^a^		0.53^a^	0.53^a^	0.62^a^	0.50^a^	0.50^a^	0.49^a^	0.40^a^	0.09^ns^
TL	0.67^a^	0.74^a^		0.53^a^	0.39^a^	0.38^a^	0.52^a^	0.28^a^	0.33^a^	0.32^a^
WH	0.67^a^	0.67^a^	0.66^a^		0.46^a^	0.35^a^	0.35^a^	0.22^a^	0.42^a^	0.19^b^
HG	0.71^a^	0.68^a^	0.75^a^	0.73^a^		0.47^a^	0.29^a^	0.41^a^	0.47^a^	−0.06^ns^
RW	0.53^a^	0.61^a^	0.63^a^	0.57^a^	0.65^a^		0.41^a^	0.34^a^	0.38^a^	0.12^ns^
RL	0.55^a^	0.59^a^	0.58^a^	0.58^a^	0.48^a^	0.46^a^		0.38^a^	0.07^ns^	0.34^a^
HL	0.59^a^	0.66^a^	0.61^a^	0.61^a^	0.61^a^	0.62^a^	0.54^a^		0.38^a^	0.21^b^
HW	0.53^a^	0.55^a^	0.54^a^	0.55^a^	0.52^a^	0.56^a^	0.41^a^	0.41^a^		0.02^ns^
EL	0.42^a^	0.35^a^	0.37^a^	0.48^a^	0.37^a^	0.26^a^	0.45^a^	0.45^a^	0.20^b^	

^a^ *P* ≤ 0.05, ^b^ *P* ≤ 0.01,* ns* non-significant

### Principal components and explained variances

From LW and nine zoometric measures of Amatepec does 5 PC were extracted which accounted for 83.3 % of the total variance (Table 4). From the analysis of Tejupilco does only 4 PC’s were extracted, which accounted for 82.4 % of the total variance.

**Table 4:** Principal components (PC), variance, partially explained variance, and cumulatively explained variance from the principal components analysis carried out for zoometric measures of local goats in Amatepec and Tejupilco, state of México

Amatepec				Tejupilco			
PC	Variance	Partially explained variance (%)	Cumulatively explained variance (%)	PC	Variance	Partially explained variance (%)	Cumulatively explained variance (%)
PC_1_	3.9	43.8	43.8	PC_1_	5.4	60.2	60.2
PC_2_	1.3	14.6	58.4	PC_2_	0.9	10.3	70.5
PC_3_	0.8	9.4	67.8	PC_3_	0.5	6.2	76.7
PC_4_	0.8	8.7	76.5	PC_4_	0.5	5.7	82.4
PC_5_	0.6	6.8	83.3	PC_5_	0.4	5.1	87.5
PC_6_	0.4	5.0	88.3	PC_6_	0.3	3.9	91.4
PC_7_	0.4	4.5	92.8	PC_7_	0.3	3.6	95.0
PC_8_	0.3	3.7	96.5	PC_8_	0.2	2.7	97.7
PC_9_	0.3	3.5	100.0	PC_9_	0.2	2.3	100.0

### Selected live weight prediction equations

Under the situation of Model 1 from the multiple linear regression analysis, the best model for Amatepec does included TL, HG, RW, and HW, with a value of R^2^ = 0.82 (Table 5), while for Tejupilco does the best model included BL, HG, HW, and EL with a value of R^2^ = 0.78, HG and HW being common measures for both populations.

**Table 5:** Multiple linear regression (stepwise) of live weight on original (model 1) and orthogonal (model 2) variables of local goats in Amatepec and Tejupilco, state of Mexico

(1) Original variables as predictors					(2) Orthogonal variables as predictors				
Variables	β_1_	SE	α	R^2^	PC	β_1_^*^	SE	α	R^2^
Amatepec									
BL	0.08	0.05	0.14	0.82	PC_1_	0.37	0.02	0.00	0.74
TL	0.18	0.07	0.01		PC_2_	−0.28	0.04	0.00	
WH	0.06	0.08	0.46		PC_3_	−0.06	0.05	0.18	
HG	0.73	0.06	0.00		PC_4_	−0.08	0.05	0.10	
RW	0.39	0.19	0.04		PC_5_	0.01	0.06	0.86	
RL	0.08	0.17	0.64		PC_6_	−0.13	0.07	0.06	
HL	−0.11	0.14	0.43		PC_7_	0.40	0.07	0.00	
HW	0.94	0.36	0.01		PC_8_	0.22	0.08	0.00	
EL	−0.06	0.13	0.67		PC_9_	−0.02	0.08	0.78	
β_0_	−49.87				γ_0_	0.00			
Tejupilco									
BL	0.34	0.06	0.00	0.76	PC_1_	0.34	0.02	0.00	0.76
TL	0.09	0.11	0.42		PC_2_	0.01	0.05	0.90	
WH	0.17	0.13	0.18		PC_3_	0.02	0.06	0.73	
HG	0.46	0.09	0.00		PC_4_	−0.04	0.06	0.51	
RW	−0.22	0.32	0.50		PC_5_	−0.19	0.07	0.00	
RL	0.25	0.17	0.14		PC_6_	−0.13	0.08	0.09	
HL	0.17	0.25	0.49		PC_7_	−0.04	0.06	0.62	
HW	0.79	0.38	0.04		PC_8_	0.05	0.09	0.58	
EL	0.43	0.15	0.01		PC_9_	0.23	0.10	0.03	
β_0_*	−54.15				γ_0_*	0.00			

*BL* body length, *TL* trunk length, *WH* withers height, *HG* hearth girth, *RW* rump width, *RL* rump length, *HL* head length, *HW* head width, *EL* ear length, *PC* principal component, *SE* standard error, *α* probability value, *R* ^*2*^ coefficient of determination

## Discussion

### Live weight and zoometric measures

The longer ears of Amatepec does, in addition to their superiority in LW and other zoometric measures, suggest the possibility that they have a higher influence of the Nubian breed than Tejupilco does, a breed that has been classified as a dual-purpose goat breed (Merlos-Brito et al. 2008). Local does of the State of Mexico have slightly lower or larger measures in various zoometrical traits than local does in different regions of México (Hernández Zepeda et al. 2002; Vargas et al. 2007), Venezuela (Pariacote et al. 2004), Argentina (Revidatti et al. 2007), and Cuba (Chacón et al. 2011). These differences can be mainly attributed to climate-related factors which generally produce changes in the quantity and quality of vegetation in different agro-ecological zones, thus leading to different ecotypes (Dossa et al. 2007). In addition, they can be also an effect of natural or artificial selection.

### Phenotypic correlations

High correlations were found between HG and BL with LW (*P* **≤** 0.01) in both populations (Table 3), which coincides with results obtained by Pariacote et al. (2004). This result suggests that producers who lack scales for weighing animals can estimate LW of their goats using either of those two zoometric measures; that is, they can use a tape rule instead of a weighing scale, a practice that is much easier to perform under field conditions.

### Principal components and explained variances

Okpeku et al. (2011) compared West African Dwarf (WAD) and Red Sokoto (RS) goats in LW and four zoometric measures by performing an APC, and extracted 2 PC in each breed. Moreover, the 2 PC extracted in WAD goats explained 94.15 % of the total variance, while the 2 PC extracted in RS goats explained 91.25 % of the total variance. Pariacote et al. (2004) performed an APC with 10 zoometric measures in local goats and extracted 10 PC, which explained 81 % of the total variation. López-Carlos et al. (2010) indicated that the first PC represent general body size, while the second PC represent body shape or conformation.

### Selected live weight prediction equations

Regarding HG, Leng et al. (2010) recommended the use of this trait as a reliable measure to predict LW in goats under field conditions, due to the fact that muscle, some fat, and bone structure contribute to its formation. Bello and Adama (2012) also found that HG was the best zoometric measure to predict LW in goats. Similar to the results of Tejupilco, Ribeiro et al. (2004) found that the best model to predict LW in local goats and their crosses included BL and HG.

Under the situation of Model 2 of the multiple regression analysis, the best model for Amatepec goats included PC_1_, PC_2_, PC_7_, and PC_8_, with a value of R^2^ = 0.76 (Table 5), while for Tejupilco goats the best model included PC_1_, PC_5_ and PC_9_, with a similar R^2^ value.

From a global analysis, considering situations of Models 1 and 2 from the multiple linear regression analysis, we conclude that the model to be used for Amatepec goats is that one that includes original variables as predictors of LW, due to their higher value of R^2^ (0.82), compared to the model that includes orthogonal variables as predictors of LW, which had a lower value of R^2^ (0.74). For Tejupilco goats the value of R^2^ was similar (0.76) under the situations of Models 1 and 2 from the multiple regression analysis, thus to predict LW of goats in this population either model can be used.

## Conclusion and implications

There are significant (*P* ≤ 0.01) differences between local does of Amatepec and Tejupilco in live weight, body length, trunk length, hearth girth, rump length and width, head length, and ear length. Trunk length, hearth girth, rump width and head width are zoometric reliable measures for predicting live weight of Amatepec local does, while for Tejupilco does body length, hearth girth, head width, and ear length are the most informative measures. Since hearth girth and head width are measures common to both populations, producers who lack scales for weighing animals in those locations are therefore recommended to utilize those measures to estimate live weight of goats with a good reliability. In addition, they will find that the use of a tape rule or graduated measuring stick signifies the saving of a considerable time and labor, especially when they are ready to sell goats based on live weight.
